# The pH-Responsive Transcription Factors YlRim101 and Mhy1 Regulate Alkaline pH-Induced Filamentation in the Dimorphic Yeast Yarrowia lipolytica

**DOI:** 10.1128/mSphere.00179-21

**Published:** 2021-05-19

**Authors:** Tao Shu, Xin-Yu He, Jia-Wen Chen, Yi-Sheng Mao, Xiang-Dong Gao

**Affiliations:** a Hubei Key Laboratory of Cell Homeostasis, College of Life Sciences, Wuhan University, Wuhan, China; b Hubei Provincial Cooperative Innovation Center of Industrial Fermentation, Wuhan, China; University of Georgia

**Keywords:** Rim101, filamentation, dimorphic transition, dimorphism, hyphal growth

## Abstract

Environmental pH influences cell growth and differentiation. In the dimorphic yeast Yarrowia lipolytica, neutral-alkaline pH strongly induces the yeast-to-filament transition. However, the regulatory mechanism that governs alkaline pH-induced filamentation has been unclear. Here, we show that the pH-responsive transcription factor Y. lipolytica Rim101 (YlRim101) is a major regulator of alkaline-induced filamentation, since the deletion of Yl*RIM101* severely impaired filamentation at alkaline pH, whereas the constitutively active Yl*RIM101^1-330^* mutant mildly induced filamentation at acidic pH. YlRim101 controls the expression of the majority of alkaline-regulated cell wall protein genes. One of these, the cell surface glycosidase gene Yl*PHR1*, plays a critical role in growth, cell wall function, and filamentation at alkaline pH. This finding suggests that YlRim101 promotes filamentation at alkaline pH via controlling the expression of these genes. We also show that, in addition to YlRim101, the Msn2/Msn4-like transcription factor Mhy1 is highly upregulated at alkaline pH and is essential for filamentation. However, unlike YlRim101, which specifically regulates alkaline-induced filamentation, Mhy1 regulates both alkaline- and glucose-induced filamentation, since the deletion of *MHY1* abolished them both, whereas the overexpression of *MHY1* induced strong filamentation irrespective of the pH or the presence of glucose. Finally, we show that YlRim101 and Mhy1 positively coregulate seven cell wall protein genes at alkaline pH, including Yl*PHR1* and five cell surface adhesin-like genes, three of which appear to promote filamentation. Together, these results reveal a conserved role of YlRim101 and a novel role of Mhy1 in the regulation of alkaline-induced filamentation in Y. lipolytica.

**IMPORTANCE** The regulatory mechanism that governs pH-regulated filamentation is not clear in dimorphic fungi except in Candida albicans. Here, we investigated the regulation of alkaline pH-induced filamentation in Yarrowia lipolytica, a dimorphic yeast distantly related to C. albicans. Our results show that the transcription factor YlRim101 and the Msn2/Msn4-like transcription factor Mhy1 are the major regulators that promote filamentation at alkaline pH. They control the expression of a number of cell wall protein genes important for cell wall organization and filamentation. Our results suggest that the Rim101/PacC homologs play a conserved role in pH-regulated filamentation in dimorphic fungi.

## INTRODUCTION

Yarrowia lipolytica is a nonconventional yeast species that has been used as a microbial cell factory for the production of multiple industrial and pharmaceutical products (1, 2). Y. lipolytica is also a dimorphic yeast that can switch its cell morphology from the oval-shaped yeast form to pseudohypha or hypha in response to environmental cues (3, 4). The development of filaments in dimorphic fungi is thought to be a foraging behavior that helps the cells to search for nutrients (5). It also plays a role in the infection of the host by pathogenic fungal species such as the human pathogen Candida albicans (6).

Environmental pH influences cell growth and differentiation in both bacteria and fungi. The shift from acidic pH to alkaline pH causes stresses to the cells by affecting the uptake of nutrients such as cations into the cell (7, 8). In fungi, the sensing and adaptation to alkaline pH are primarily carried out by the Rim101/PacC signaling pathway (9, 10). The cell surface proteins Rim21 and Dfg16 of this pathway senses alkaline pH in the environment and elicit a series of signal transduction events that activate Rim101/PacC, a zinc finger transcription factor, via other Rim/Pal proteins. The activation of Rim101/PacC involves the proteolytic removal of the inhibitory C-terminal region, which allows Rim101/PacC to enter the nucleus, bind to the promoters of target genes, and activate or repress their expression. The Rim101/PacC signaling pathway was initially identified in the yeast Saccharomyces cerevisiae and the filamentous fungus Aspergillus nidulans (11, 12) but was later found to be well conserved in other fungi (9, 13). This pathway controls a number of pH responses, including sporulation and haploid invasive growth in S. cerevisiae and the production of alkaline proteases and phosphatases in A. nidulans.

pH is an important environmental factor that affects the yeast-to-filament transition in dimorphic fungi. In the dimorphic yeasts C. albicans and Y. lipolytica, acidic pH promotes yeast-form growth, whereas neutral-alkaline pH induces filamentation (4, 14). However, in other dimorphic fungi, such as Ustilago maydis and *Trichosporon cutaneum*, acidic pH induces filamentation, whereas neutral-alkaline pH promotes yeast-form growth (15, 16). The regulatory mechanism that governs pH-regulated filamentation is not well understood except in C. albicans. Studies in C. albicans showed that the Rim101/PacC homolog, C. albicans Rim101 (CaRim101), plays a crucial role in the regulation of filamentation at neutral-alkaline pH (17). CaRim101 positively regulates the expression of Ca*PHR1*, a cell surface glycosidase gene important for growth, cellular morphogenesis, and filamentation at neutral-alkaline pH (18, 19). CaRim101 also positively regulates the expression of cell surface adhesin genes, such as *HWP1* and *HYR1* (8), which are associated with hyphal formation (20, 21).

Although CaRim101 plays an important role in the control of pH-regulated filamentation in C. albicans, it is not known whether the Rim101/PacC homologs also control pH-regulated filamentation in other dimorphic fungi. In this study, we show that Y. lipolytica Rim101 (YlRim101) shares a conserved function with C. albicans CaRim101 in the regulation of pH-regulated filamentation. In addition, we reveal a novel role of the Msn2/Msn4-like transcription factor Mhy1 in the regulation of pH-regulated filamentation.

## RESULTS

### YlRim101 is a major regulator of alkaline-induced filamentation.

The Rim101/PacC signaling pathway plays an important role in the adaptation to alkaline pH in fungi (9, 13). In the dimorphic yeasts C. albicans and Y. lipolytica, alkaline pH strongly induces the yeast-to-filament transition (4, 14). While CaRim101 plays an important role in the regulation of filamentation at alkaline pH in C. albicans (17), previous studies suggested that YlRim101 is not required for filamentation in Y. lipolytica (22, 23). Thus, we deleted the Yl*RIM101* (*YALI0B13640*) gene in the wild-type strain PO1a and reexamined the role of YlRim101 in alkaline-induced filamentation.

**FIG 1:**
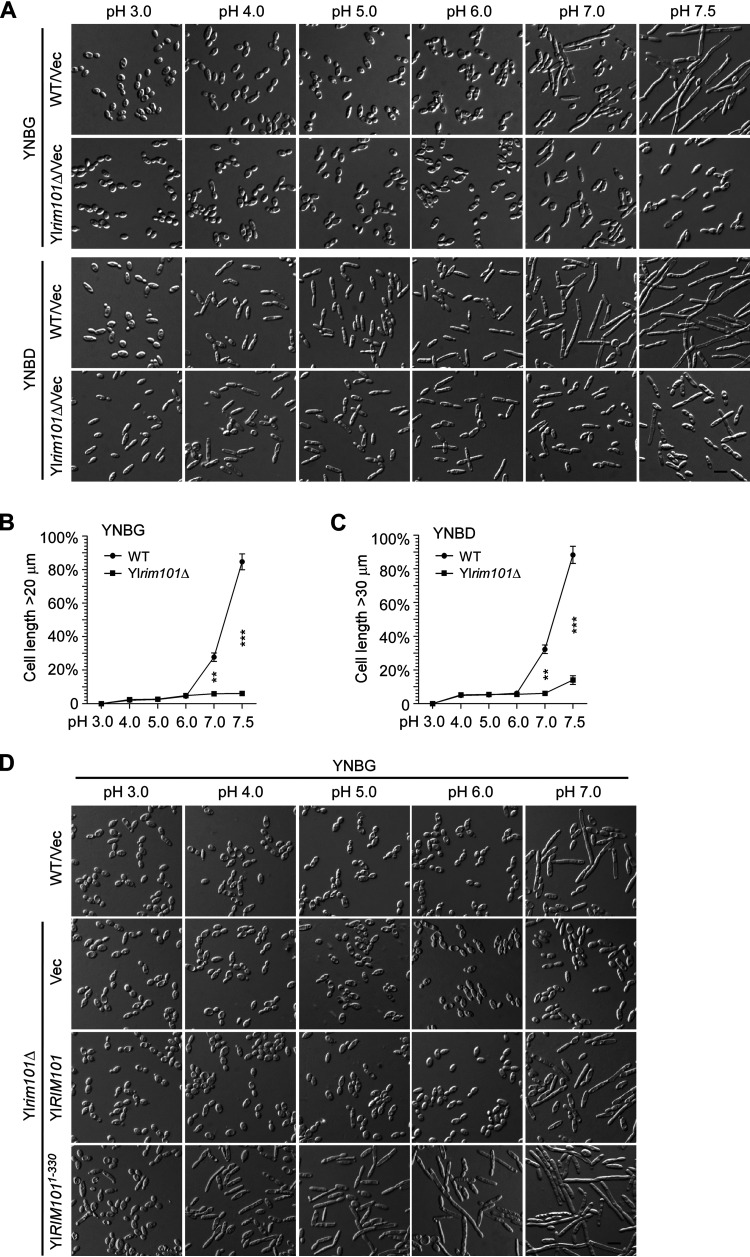
YlRim101 positively regulates alkaline-induced filamentation. (A) Cells of the wild-type (WT) and Yl*rim101*Δ strains carrying the plasmid vector pINA445 (Vec) were grown in liquid YNBG (glycerol) and YNBD (glucose) media buffered at pHs ranging from 3.0 to 7.5 at 30°C. (B and C) Cells as in panel A were measured for cell length. The percentages of cells longer than 20 μm (B, YNBG medium) and 30 μm (C, YNBD medium) are shown (*n *> 600 cells). The mother cell and the bud that it carries were counted as one cell. Statistically significant differences are indicated by asterisks (**, *P* < 0.01; ***, *P* < 0.001). (D) Cells of the Yl*rim101*Δ mutant carrying plasmid pINA445, pINA445-YlRIM101, or pINA445-YlRIM101^1-330^ as well as the wild-type strain carrying pINA445 were grown in YNBG medium buffered at pHs from 3.0 to 7.0 at 30°C. Bars, 10 μm.

Y. lipolytica can efficiently utilize both glycerol and glucose. When grown in glycerol medium, the wild-type strain PO1a grew in the oval-shaped yeast form at acidic pH (pH 3.0 to pH 6.0), but the cells became markedly elongated at neutral pH (pH 7.0) and formed long filaments at slightly alkaline pH (pH 7.5) (Fig. 1A, top row). Twenty-eight percent and 85% of wild-type cells were longer than 20 μm at pH 7.0 and pH 7.5, respectively (Fig. 1B). In contrast, the cells of the Yl*rim101*Δ mutant were just slightly elongated at pH 7.0 but did not form filaments at pH 7.5 (Fig. 1A, second row). Moreover, only 6% of the Yl*rim101*Δ cells were longer than 20 μm at pH 7.0, and this number did not increase at pH 7.5 (Fig. 1B). This finding suggests that YlRim101 is crucial for alkaline-induced filamentation. Reintroduction of the Yl*RIM101* gene into the Yl*rim101*Δ mutant restored filament formation at pH 7.5 (see Fig. S1 in the supplemental material), indicating that the filamentation defect was caused by Yl*RIM101* deletion.

10.1128/mSphere.00179-21.1 FIG S1 Complementation of the filamentation defect of the Yl*rim101*Δ mutant. Cells of the Yl*rim101*Δ mutant carrying plasmid pINA445, pINA445-YlRIM101, pINA445-YlRIM101^1-419^, or pINA445-YlRIM101^1-330^ as well as the wild-type strain carrying pINA445 were grown in YNBG medium buffered at pH 7.5 at 30°C. Strength in the complementation capacity of the filamentation defect by the C-terminally truncated YlRim101 mutants is indicated from strong (++++) to weak (+). Bar, 10 μm. Download FIG S1, TIF file, 0.3 MB. Copyright © 2021 Shu et al. 2021 Shu et al. https://creativecommons.org/licenses/by/4.0/ This content is distributed under the terms of the Creative Commons Attribution 4.0 International license.

When grown in glucose medium, the wild-type strain displayed a markedly elongated morphology at acidic pH (pH 4.0 to pH 6.0), presumably due to glucose-stimulated weak filamentation (see Discussion). Long filaments were formed at pH 7.0 and pH 7.5 (Fig. 1A, third row). Thirty-two percent and 88% of wild-type cells were longer than 30 μm at pH 7.0 and pH 7.5, respectively (Fig. 1C). In contrast, the Yl*rim101*Δ mutant retained the elongated morphology but did not form long filaments at pH 7.0 and pH 7.5 (Fig. 1A, bottom row). Moreover, only 6% and 14% of the Yl*rim101*Δ cells were longer than 30 μm at pH 7.0 and pH 7.5, respectively (Fig. 1C). This finding supports the view that YlRim101 is crucial for filamentation at alkaline pH. The Yl*rim101*Δ cells still weakly responded to alkaline stimulation, since the percentage of Yl*rim101*Δ cells longer than 30 μm at pH 7.5 was slightly higher than that of pH 7.0 (Fig. 1C). The difference is statistically significant (*P* < 0.05).

Next, we examined whether the active form of YlRim101 induces filamentation in glycerol medium at acidic pH, where filamentation normally does not occur. Upon upstream signaling at alkaline pH, Rim101/PacC undergoes enzymatic cleavage to remove the inhibitory C-terminal region (9, 13). The C-terminally truncated form of Rim101/PacC is thought to be the active form for function. Although the cleavage site has not been determined for YlRim101, a previous study showed that the C-terminally truncated alleles Yl*RIM101-1119* (encodes YlRim101^1-330^) and Yl*RIM101-5* (encodes YlRim101^1-419^) induced *XPR2-lacZ* expression at pH 4.0, whereas wild-type Yl*RIM101* did not (24), indicating that YlRim101^1-330^ and YlRim101^1-419^ are constitutively active. We observed that the expression of YlRim101^1-330^ and YlRim101^1-419^ mutants in the Yl*rim101*Δ mutant efficiently restored filament formation in glycerol medium at pH 7.5 (see Fig. S1 in the supplemental material). Thus, they can be used to mimic the activated form of YlRim101.

The expression of the YlRim101^1-330^ mutant in the Yl*rim101*Δ strain grown in glycerol medium caused cell elongation at pHs from 3.0 to 5.0 and the formation of some long filaments at pH 6.0 (Fig. 1D). Like YlRim101^1-330^, the YlRim101^1-419^ mutant displayed a similar effect at acidic pH (data not shown). In contrast, the expression of wild-type YlRim101 in the Yl*rim101*Δ strain did not cause cell elongation at pHs from 3.0 to 6.0 (Fig. 1D). Moreover, the overexpression of the wild-type YlRim101 in the wild-type strain under the control of the strong Yl*TEF1* promoter still did not cause cell elongation in glycerol medium at pH 4.0 (data not shown). This finding indicates that the constitutively active form of YlRim101 is capable of causing mild filamentation at acidic pH.

### YlRim101 controls the expression of the majority of alkaline-regulated cell wall protein genes.

To explore how YlRim101 regulates alkaline-induced filamentation, we wanted to identify the genes that are susceptible to alkaline induction. To this end, we performed transcriptome analysis of the wild-type strain PO1a grown in glycerol medium buffered at acidic pH (pH 4.0) and slightly alkaline pH (pH 7.5) by transcriptome sequencing (RNA-Seq). Our results indicated that a total of 1,593 genes were significantly differentially expressed (≥2-fold, *P* < 0.05) at pH 7.5 compared to that at pH 4.0 (the full set of data will be published elsewhere). Of these, 621 genes were upregulated, whereas 972 genes were downregulated at pH 7.5. A total of 124 genes were highly upregulated (≥5-fold), whereas 300 genes were highly downregulated (≥5-fold).

The cell wall plays an important role in filamentation. It is required for the maintenance of hyphal growth. Therefore, the cell wall protein genes that were significantly differentially expressed (≥2-fold, *P* < 0.05) at pH 7.5, particularly those that were upregulated, drew our attention. A total of 41 cell wall protein genes were upregulated at pH 7.5 (Table 1, Table S1). Fifteen of them were highly upregulated (≥5-fold). These genes encode putative cell wall structural proteins, such as the ones similar to S. cerevisiae Cwp1, cell surface enzymes involved in cell wall biosynthesis such as YlPhr1 and YlCrh12, and cell surface proteins similar to S. cerevisiae Flo11 or C. albicans Hyr1. All these proteins appear to possess a signal peptide. The majority of them are putative glycosylphosphatidylinositol (GPI)-anchored cell surface proteins, whereas several proteins are transmembrane proteins. There were also 26 cell wall protein genes that were downregulated at pH 7.5 (Table 1, Table S1). Sixteen of them were highly downregulated (≥5-fold). These genes encode putative cell wall structural proteins, such as the ones similar to S. cerevisiae Pir1 and Cwp1, cell surface enzymes similar to S. cerevisiae Yps3 and Dcw1, the cell surface glycosidase YlPhr2, and cell surface proteins similar to S. cerevisiae Flo11 or C. albicans Hyr1.

10.1128/mSphere.00179-21.2 TABLE S1 RNA-Seq data for cell wall protein genes in WT (pH 7.5) versus WT (pH 4.0) datasets. Download Table S1, XLS file, 0.1 MB. Copyright © 2021 Shu et al. 2021 Shu et al. https://creativecommons.org/licenses/by/4.0/ This content is distributed under the terms of the Creative Commons Attribution 4.0 International license.

**TABLE 1:** The alkaline-upregulated and alkaline-downregulated cell wall protein genes identified by RNA-Seq^*a*^

Direction of regulation	YlRim101-regulated genes	Non-YlRim101-regulated genes
Up		
High (≥5-fold), 15 genes	*YALI0F26565* (U2), *YALI0E22286* (U4), ***YALI0A00176*** (U6), *PHR1* (*YALI0D04851* U8), ***YALI0C11165*** (U15), ***YALI0C23452*** (U19), *YALI0E26125* (U22), *YALI0A21373* (U26), *YALI0D09185* (U33), *YALI0E01210* (U61), ***YALI0F19030*** (U74), ***YALI0B18194*** (U103), *YALI0A17919* (U111), *YALI0D17248* (U120)	*YALI0E19426* (U113)
Low (≥2-fold), 26 genes	*CWP*1 (*YALI0E18788*, U158), *CRH12* (*YALI0E24673*, U166), *CHS2* (*YALI0B16324*, U204), *YALI0D17270* (U338), ***YALI0A11198*** (U488), ***YALI0C08473*** (U550), ***YALI0F00990*** (U573), ***YALI0F10901*** (U578)	*YALI0D00154* (U138), *CWH43* (*YALI0E33473*, U149), *YALI0E20823* (U150), *PSA1* (*YALI0C06490*, U177), *YALI0F21428* (U203), *DFG5* (*ALI0F18722*, U205), *YALI0E22088* (U229)^*b*^, *CRH11* (*YALI0C09680*, U232), *UTR2* (*YALI0B15510*, U320), *YALI0F01925* (U322), *UAP1* (*YALI0E03146*, U353), *CHS5* (*YALI0E16170*, U372), *YALI0B03564* (U376), *KRE6* (*YALI0C14190*, U434), *YALI0E10175* (U480), *CHS3* (*YALI0C24354*, U482), *CWH41* (*YALI0F14927*, U526), *YALI0E22374* (U564)
Down		
High (≥5-fold), 16 genes	*PIR2* (*YALI0C02981*, D9), *PIR1* (*YALI0B20306*, D23), *YALI0F18282* (D24), *YALI0C22836g* (D40), *YALI0A13013* (D91), *YALI0A20438* (D115), *YALI0E31108* (D163), *YALI0A02002* (D166), *YALI0B07403* (D167), *EXG1* (*YALI0F05390*, D231), *PHR2* (*YALI0D06039*, D232), *YALI0C14938* (D247), *YALI0C10135* (D251), *YALI0C01411* (D260)	*YALI0A08800* (D196), *YALI0D01331* (D277)
Low (≥2-fold), 10 genes	*YALI0E33891* (D720)	*YALI0B20174* (D398), ***YALI0E22572*** (D434), ***YALI0E18722*** (D482), ***YALI0B09867*** (D523), *YALI0F21857* (D579), *CDA1* (*YALI0F30833*, D581), *YALI0F09163* (D730), *YALI0C12980* (D835), ***YALI0C13970*** (D839)

a Numbers in parentheses indicate each gene’s ranking in the full list of the RNA-Seq data set. Genes that showed at least 2-fold changes (*P *< 0.05) in the Yl*rim101*Δ mutant compared to that in the wild-type strain are defined as YlRim101 regulated. Genes that encode proteins that share similarities to the cell surface adhesin S. cerevisiae Flo11 or C. albicans Hyr1 are in boldface. *YALI0D09185*, which encodes a protein that shares weak similarity to the S. cerevisiae adhesin Aga1 but lacks an identifiable GPI modification site, is underlined.

b *YALI0E22088* is classified as non-YlRim101 regulated, because its transcription did not decrease but rather increased in the Yl*rim101*Δ mutant, and the extent of the increase is marginal (2.0-fold, *P *= 0.03).

**FIG 2:**
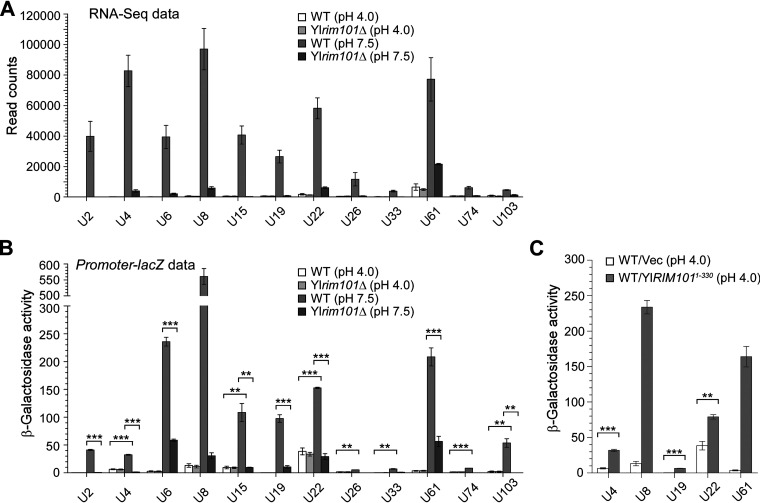
YlRim101 controls the expression of the majority of alkaline-upregulated cell wall protein genes. (A and B) Transcriptional activities of the top 12 highly alkaline-upregulated cell wall protein genes U2-U103 in cells of the wild-type (WT) and Yl*rim101*Δ strains carrying pINA445 (A) or pINA445-P_Gene_-*lacZ* (B) grown in YNBG medium buffered at pH 4.0 and pH 7.5 at 30°C. The RNA-Seq read counts (A) and the β-galactosidase activities of *promoter-lacZ* (B) are shown. Statistically significant differences are indicated by the asterisks (**, *P* < 0.01; ***, *P* < 0.001). (C) β-Galactosidase activities of *promoter-lacZ* for the indicated cell wall protein genes in cells of the wild-type strain carrying pINA445-P_Gene_-*lacZ*/pINA443 or pINA445-P_Gene_-*lacZ*/pINA443-YlRIM101^1-330^ grown in YNBG medium buffered at pH 4.0 at 30°C.

We also examined the transcriptome of the Yl*rim101*Δ mutant at pH 7.5 by RNA-Seq and compared it with that of the wild-type strain. Our results showed that 22 of the 41 alkaline-upregulated cell wall protein genes (54%) exhibited a significant reduction in read counts (≥2-fold, *P* < 0.05) in the Yl*rim101*Δ mutant, indicating that they were YlRim101 regulated. Among the 15 highly alkaline-upregulated cell wall protein genes, 14 of them (93%) were YlRim101 regulated (Table 1, Table S2; Fig. 2A depicts the top 12 genes). This finding suggests that YlRim101 is important for the upregulation of the majority of alkaline-upregulated cell wall protein genes, particularly the highly alkaline-upregulated ones. We monitored the top 12 alkaline-upregulated cell wall protein genes for their transcriptional activities in the Yl*rim101*Δ mutant by using *promoter-lacZ* reporters. They were all markedly reduced in the Yl*rim101*Δ mutant compared to those in the wild-type strain (Fig. 2B). Remarkably, the expression of the constitutively active YlRim101^1-330^ mutant in the wild-type strain at pH 4.0 significantly upregulated the transcription levels of five of the 12 genes, *YALI0E22286* (U4), Yl*PHR1* (U8), *YALI0C23452* (U19), *YALI0E26125* (U22), and *YALI0E01210* (U61) (Fig. 2C). These results indicate that YlRim101 positively regulates a subset of alkaline-upregulated cell wall protein genes, which may play roles in cell wall organization and filamentation.

10.1128/mSphere.00179-21.3 TABLE S2 RNA-Seq data for cell wall protein genes in Yl*rim101*Δ (pH 7.5) versus WT (pH 7.5) datasets. Download Table S2, XLS file, 0.1 MB. Copyright © 2021 Shu et al. 2021 Shu et al. https://creativecommons.org/licenses/by/4.0/ This content is distributed under the terms of the Creative Commons Attribution 4.0 International license.

Among the 26 alkaline-downregulated cell wall protein genes, 15 of them (58%) were YlRim101 regulated (≥2-fold, *P* < 0.05). Among the 16 highly alkaline-downregulated genes, 14 of them (87%) were YlRim101 regulated (Table 1, Table S2). This finding suggests that YlRim101 is also important for the downregulation of the majority of alkaline-downregulated cell wall protein genes.

We found that all the 41 alkaline-upregulated and the 26 alkaline-downregulated cell wall proteins did not exhibit differential expression (≥2-fold, *P* < 0.05) in the wild-type and Yl*rim101*Δ strains at pH 4.0 (Table S3). This finding suggests that YlRim101 does not regulate the expression of these genes at acidic pH.

10.1128/mSphere.00179-21.4 TABLE S3 RNA-Seq data for cell wall protein genes in Yl*rim101*Δ (pH 4.0) versus WT (pH 4.0) datasets. Download Table S3, XLS file, 0.1 MB. Copyright © 2021 Shu et al. 2021 Shu et al. https://creativecommons.org/licenses/by/4.0/ This content is distributed under the terms of the Creative Commons Attribution 4.0 International license.

### The YlRim101-regulated cell surface glycosidase genes Yl*PHR1* and Yl*PHR2* are required for growth, cell wall function, and filamentation.

**FIG 3:**
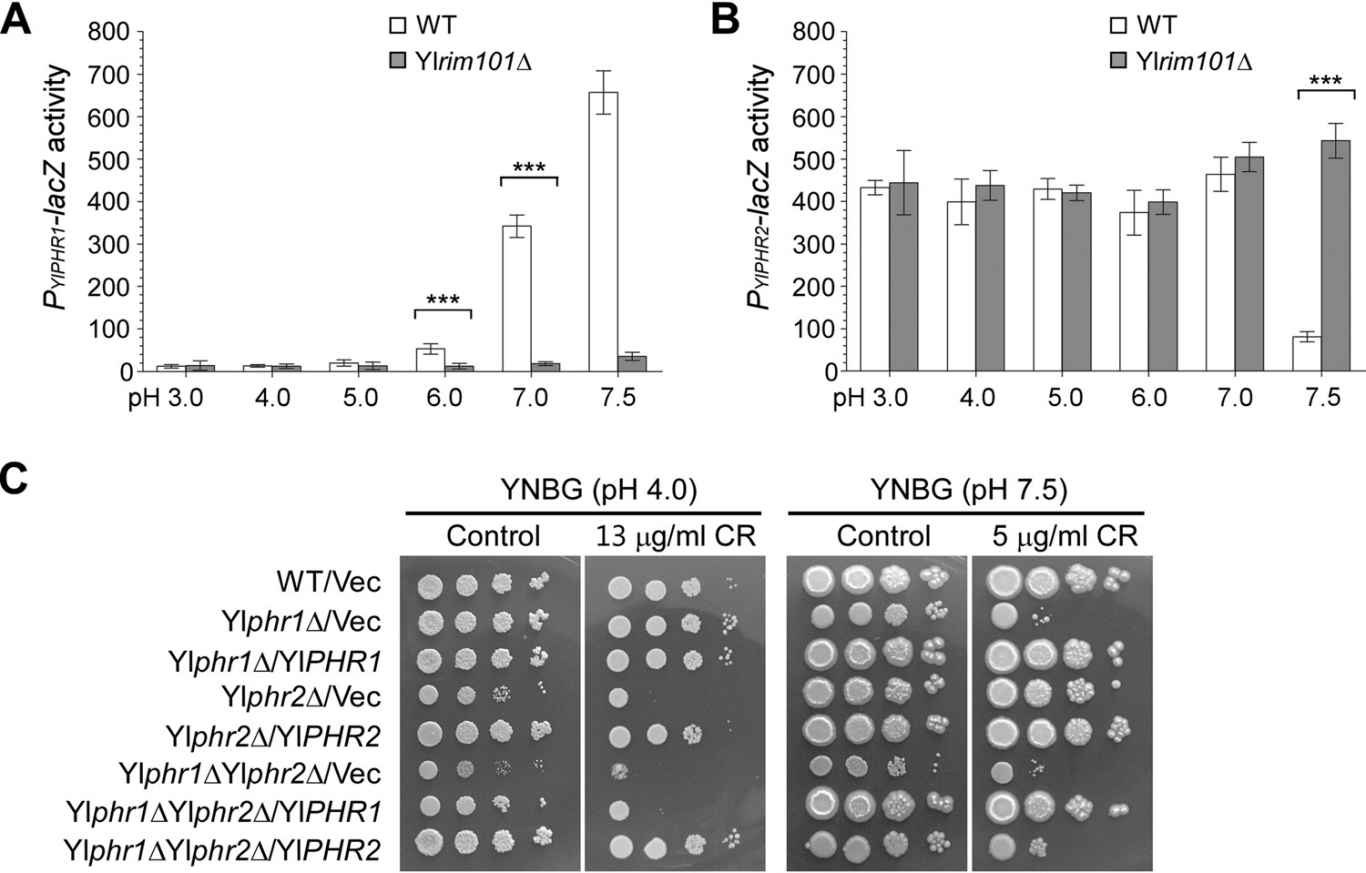
Cell surface glycosidase genes Yl*PHR1* and Yl*PHR2* are alkaline- and YlRim101-regulated and are required for normal growth and cell wall function. (A and B) β-Galactosidase activities of *P_YlPHR1_-lacZ* (A) and *P_YlPHR2_-lacZ* (B) in cells of the wild-type (WT) and Yl*rim101*Δ strains grown in YNBG medium buffered at the indicated pH. (C) Cells of the wild-type (WT), Yl*phr1*Δ, Yl*phr2*Δ, and Yl*phr1*Δ Yl*phr2*Δ strains carrying plasmid pINA445 (Vec), pINA445-YlPHR1, or pINA445-YlPHR2 were spotted at 1:10 serial dilution on YNBG plates buffered at pH 4.0 and pH 7.5 and supplemented with or without Congo red (CR). Pictures were taken after being grown at 30°C for 2 days.

Yl*PHR1* and its homolog, Yl*PHR2*, encode proteins that are highly similar to S. cerevisiae Gas1 and C. albicans CaPhr1 and CaPhr2, which are GPI-anchored cell surface 1,3-β-glucanosyltransferases necessary for cell wall organization and cellular morphogenesis (18, 25, 26). Previous studies have shown that Yl*PHR1* and Yl*PHR2* are pH-responsive genes and are transcriptionally regulated by YlRim101 in an inverted pattern similar to that of C. albicans Ca*PHR1* and Ca*PHR2*, respectively (27–29). We obtained a similar result by RNA-Seq and *promoter-lacZ* analyses. Yl*PHR1* (U8) was highly upregulated at pH 7.0 and pH 7.5 (Table 1, Fig. 3A), whereas Yl*PHR2* was highly downregulated at pH 7.5 (Table 1, Fig. 3B). In addition, YlRim101 is required for both the upregulation of Yl*PHR1* at pH 7.0 and pH 7.5 as well as the downregulation of Yl*PHR2* at pH 7.5 (Fig. 3A and B).

Since the cellular roles of Yl*PHR1* and Yl*PHR2* have not been characterized previously, we generated Yl*phr1*Δ, Yl*phr2*Δ, and Yl*phr1*Δ Yl*phr2*Δ mutants and examined the growth and cell morphology. We observed that the Yl*phr1*Δ and Yl*phr2*Δ mutants exhibited slower growth and increased sensitivity to the cell wall-perturbing agent Congo red compared to the wild-type strain at pH 7.5 and pH 4.0, respectively, whereas the Yl*phr1*Δ Yl*phr2*Δ double mutant exhibited the same defects at both pH 4.0 and pH 7.5 (Fig. 3C). This finding suggests that Yl*PHR1* and Yl*PHR2* are required for normal growth and cell wall function at alkaline pH and acidic pH, respectively, similar to C. albicans Ca*PHR1* and Ca*PHR2* (18, 25).

**FIG 4:**
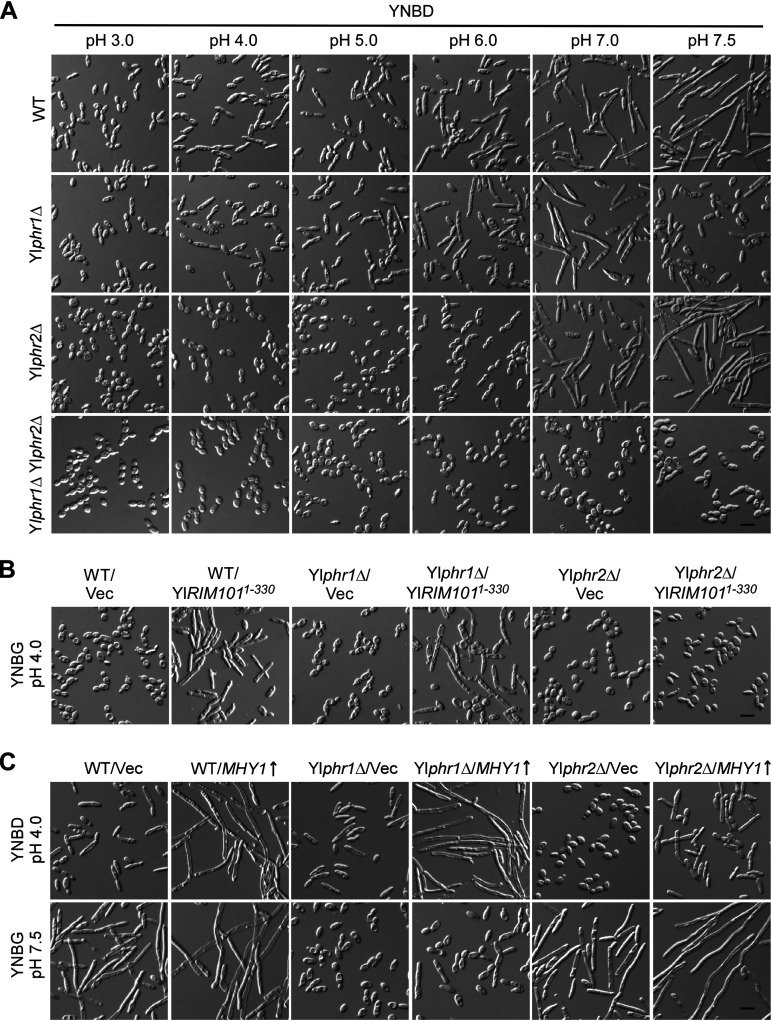
Yl*PHR1* and Yl*PHR2* are required for filamentation. (A) Cells of the wild-type (WT), Yl*phr1*Δ, Yl*phr2*Δ, and Yl*phr1*Δ Yl*phr2*Δ strains carrying pINA445 were grown in YNBD medium buffered at pHs ranging from 3.0 to 7.5 at 30°C. (B) Cells of the wild-type, Yl*phr1*Δ, and Yl*phr2*Δ strains carrying pINA445 (Vec) or pINA445-YlRIM101^1-330^ were grown in YNBG medium buffered at pH 4.0 at 30°C. (C) Cells of the wild-type, Yl*phr1*Δ, and Yl*phr2*Δ strains carrying pYL13 (Vec) or pYL13-MHY1 were grown in YNBD and YNBG media buffered at pH 4.0 and pH 7.5, respectively, at 30°C. Bars, 10 μm.

The Yl*phr1*Δ mutant exhibited normal cell morphology in YNBD medium at pHs ranging from 3.0 to 7.0. However, it failed to form long filaments at pH 7.5 (Fig. 4A, second row), indicating that Yl*PHR1* is required for cellular morphogenesis specifically at alkaline pH, i.e., alkaline-induced filamentation. In contrast to the Yl*phr1*Δ mutant, the Yl*phr2*Δ mutant exhibited normal filamentation at pH 7.0 and pH 7.5. However, at acidic pH (pH 3.0 to pH 6.0), the Yl*phr2*Δ mutant exhibited a round cell morphology (Fig. 4A, third row), which is different from the elongated cell morphology of the wild-type strain, indicating that Yl*PHR2* is required for cellular morphogenesis specifically at acidic pH. The Yl*phr1*Δ Yl*phr2*Δ mutant did not exhibit the elongated cell morphology at pHs ranging from 3.0 to 6.0 or formed filaments at pH 7.0 and pH 7.5 (Fig. 4A, bottom row), suggesting that Yl*PHR1* and Yl*PHR2* are the two major 1,3-β-glucanosyltransferase genes in the cells. We noticed that the Yl*phr1*Δ mutant failed to form long filaments at pH 7.5 but formed them at pH 7.0 (Fig. 4A, second row). This is likely due to the functional compensation by YlPhr2, which is still highly expressed at pH 7.0.

We showed earlier that the constitutively active YlRim101^1-330^ mutant induced filamentation at acidic pH (Fig. 1D). This effect can still be seen in the Yl*phr1*Δ mutant but was severely impaired in the Yl*phr2*Δ mutant at pH 4.0 (Fig. 4B), suggesting that Yl*PHR2* is important for filamentation at acidic pH. The overexpression of *MHY1* induced the formation of long filaments in the wild-type strain. However, this effect was compromised in the Yl*phr2*Δ mutant at pH 4.0 and in the Yl*phr1*Δ mutant at pH 7.5 (Fig. 4C), supporting the view that Yl*PHR1* and Yl*PHR2* are required for filament formation at alkaline pH and acidic pH, respectively.

### Yl*RIM101* and *MHY1* are the two major transcription factor genes highly upregulated at alkaline pH.

In addition to the cell wall protein genes, there were 25 transcription factor genes that exhibited significant differential expression (≥2-fold, *P* < 0.05) at pH 7.5 compared to that at pH 4.0, as shown by RNA-Seq (Table 2, Table S4). Of these, 11 genes were upregulated, whereas 14 genes were downregulated at pH 7.5. Three genes were highly upregulated (≥5-fold), whereas five genes were highly downregulated (≥5-fold).

**TABLE 2:** The alkaline-upregulated and alkaline-downregulated transcription factor genes identified by RNA-Seq^*a*^

Direction of regulation	YlRim101-regulated genes	Non-YlRim101-regulated genes
Up		
High (≥5-fold), 3 genes	*MHY1* (*YALI0B21582*, U46), *YALI0A12925* (U49), *RIM101* (*YALI0B13640*, U122)	
Low (≥2-fold), 8 genes	*ACE2* (*YALI0E16973*, U127), *YALI0F25113* (U165), *YALI0C09482* (U220), *YALI0C08327* (U300)	*YALI0E20449* (U131), *YALI0B13354* (U218), *YALI0E29271* (U321), *TEC1* (*YALI0F15169*, U601)
Down		
High (≥5-fold), 5 genes	*YALI0D15664* (D101), *YALI0B05478* (D134), *YALI0F03157* (D298)	*YALI0E18161* (D212), *YALI0D20482* (D280)
Low (≥2-fold), 9 genes	*YALI0E23518* (D661), *PPR1* (*YALI0B09713*, D822)	*YALI0B00660* (D527), *YALI0D24167* (D616), *YALI0C13178* (D732), *YALI0A16841* (D906), *YALI0D09625* (D934), *YALI0D20394* (D966), *YALI0F16599* (970)

a Numbers in parentheses indicate each gene’s ranking in the full list of the RNA-Seq data set. Genes that showed at least 2-fold changes (*P *< 0.05) in the Yl*rim101*Δ mutant compared to that in the wild-type strain are defined as YlRim101 regulated.

10.1128/mSphere.00179-21.5 TABLE S4 RNA-Seq data for transcription factor genes in WT (pH 7.5) versus WT (pH 4.0) datasets. Download Table S4, XLS file, 0.04 MB. Copyright © 2021 Shu et al. 2021 Shu et al. https://creativecommons.org/licenses/by/4.0/ This content is distributed under the terms of the Creative Commons Attribution 4.0 International license.

**FIG 5:**
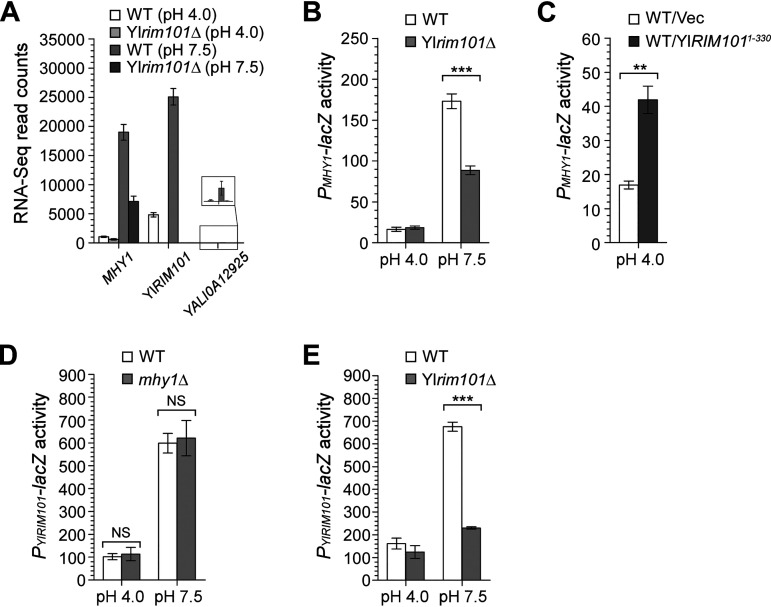
Yl*RIM101* and *MHY1* are the two major transcription factor genes upregulated at alkaline pH. (A) RNA-Seq read counts of the transcription factor genes *MHY1*, Yl*RIM101*, and *YALI0A12925*. Cells of the wild-type (WT) and Yl*rim101*Δ strains carrying pINA445 were grown in YNBG medium buffered at pH 4.0 and pH 7.5 at 30°C. For *YALI0A12925*, a blown-up bar graph is shown in the box, because its read counts are too low (less than 40) to be seen. (B) β-Galactosidase activities of *P_MHY1_-lacZ* in cells of the wild-type and Yl*rim101*Δ strains carrying pINA445-P_MHY1_-*lacZ* grown in YNBG medium buffered at pH 4.0 and pH 7.5 at 30°C. (C) β-Galactosidase activities of *P_MHY1_-lacZ* in cells of the wild-type strain carrying pINA445-P_MHY1_-*lacZ*/pINA443 (Vec) or pINA445-P_MHY1_-*lacZ*/pINA443-YlRIM101^1-330^ grown in YNBG medium buffered at pH 4.0 at 30°C. (D) β-Galactosidase activities of *P_YlRIM101_-lacZ* in cells of the wild-type and *mhy1*Δ strains carrying pINA445-P_YlRIM101_-*lacZ* grown in YNBG medium buffered at pH 4.0 and pH 7.5 at 30°C. (E) β-Galactosidase activities of *P_YlRIM101_-lacZ* in cells of the wild-type and Yl*rim101*Δ strains carrying pINA445-P_YlRIM101_-*lacZ* grown in YNBG medium buffered at pH 4.0 and pH 7.5 at 30°C. Statistically significant differences are indicated by the asterisks (**, *P* < 0.01; ***, *P < *0.001). NS, not statistically significant.

The two highly alkaline-upregulated transcription factor genes Yl*RIM101* and *MHY1* drew our attention, since they both regulate filamentation. *MHY1* encodes an Msn2/Msn4-like zinc finger transcription factor that plays a key role in filamentation (30, 31). It is not surprising to find Yl*RIM101* on the list, since Yl*RIM101* is known to be alkaline induced (24). The identification of *MHY1* as an alkaline-induced gene is unexpected. Yl*RIM101* and *MHY1* appear to be the two major transcription factor genes upregulated at alkaline pH, since they were among the three highly alkaline-induced transcription factor genes (Fig. 5A). Additionally, they exhibited the highest RNA-Seq read counts at pH 7.5 among all 11 alkaline-upregulated genes (Table S4).

Both RNA-Seq and *promoter-lacZ* data indicate that *MHY1* is an alkaline-induced gene, since its transcription was highly upregulated at alkaline pH (Fig. 5A and B). Our results also showed that the level of *MHY1* transcription was reduced about 2-fold in the Yl*rim101*Δ mutant at pH 7.5 (Fig. 5A and B, and Table S5). Moreover, the level of *MHY1* transcription increased 2.5-fold upon the expression of the constitutively active YlRim101^1-330^ mutant at pH 4.0 (Fig. 5C). These results indicate that YlRim101 partially regulates *MHY1*. Since a significant portion of *MHY1* upregulation was retained in the Yl*rim101*Δ mutant at pH 7.5, the upregulation of *MHY1* may also involve a YlRim101-independent mechanism. We found that Mhy1 does not regulate Yl*RIM101* (Fig. 5D).

10.1128/mSphere.00179-21.6 TABLE S5 RNA-Seq data for transcription factor genes in Yl*rim101*Δ (pH 7.5) versus WT (pH 7.5) datasets. Download Table S5, XLS file, 0.04 MB. Copyright © 2021 Shu et al. 2021 Shu et al. https://creativecommons.org/licenses/by/4.0/ This content is distributed under the terms of the Creative Commons Attribution 4.0 International license.

In addition to *MHY1*, YlRim101 also regulates Yl*RIM101* itself. The alkaline-induced expression of Yl*RIM101* was severely impaired in the Yl*rim101*Δ mutant, as shown by the *P_YlRIM101_-lacZ* reporter (Fig. 5E), in agreement with a previous report (24).

### Mhy1 regulates both alkaline- and glucose-induced filamentation.

**FIG 6:**
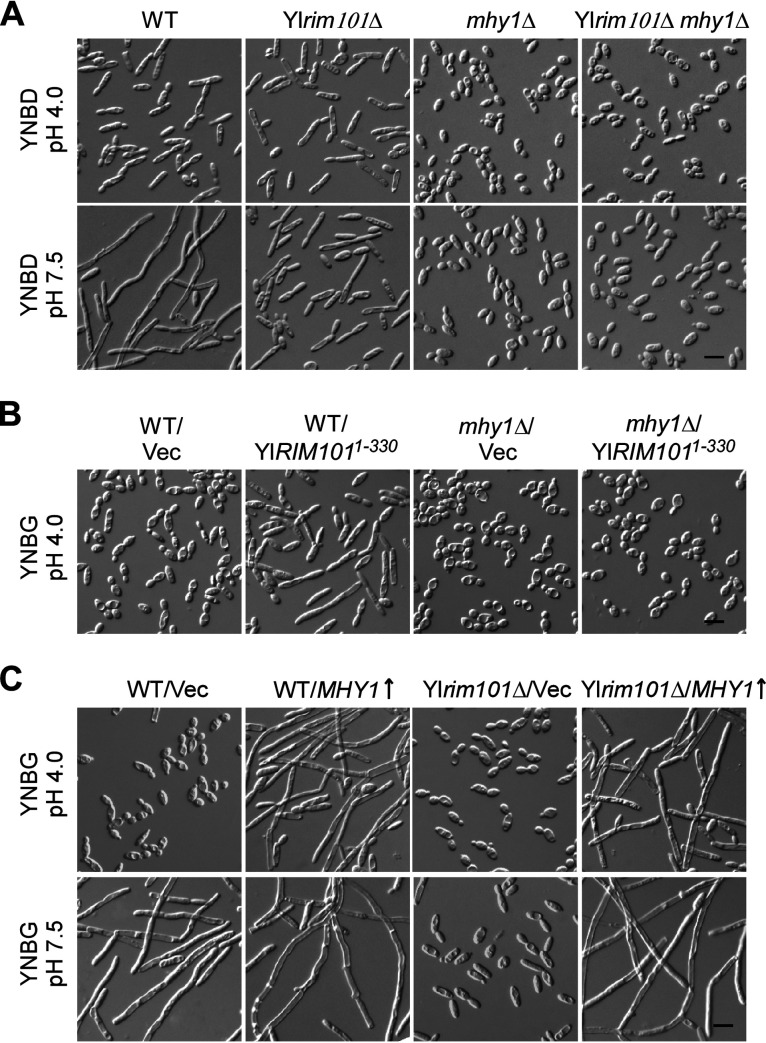
Mhy1 regulates both alkaline- and glucose-induced filamentation. (A) Cells of wild-type (WT), Yl*rim101*Δ, *mhy1*Δ, and Yl*rim101*Δ *mhy1*Δ strains carrying pINA445 were grown in YNBD medium buffered at pH 4.0 and pH 7.5 at 30°C. (B) Cells of the wild-type and *mhy1*Δ strains carrying pINA445 (Vec) or pINA445-YlRIM101^1-330^ were grown in YNBG medium buffered at pH 4.0 at 30°C. (C) Cells of the wild-type and Yl*rim101*Δ strains carrying pYL13 or pYL13-MHY1 were grown in YNBG medium buffered at pH 4.0 and pH 7.5 at 30°C. Bars, 10 μm.

We observed that the *mhy1*Δ mutant exhibited an oval-shaped yeast-form morphology at both pH 4.0 and pH 7.5, even in the filamentation-favoring glucose medium. It neither exhibited an elongated morphology at pH 4.0 nor formed any filaments at pH 7.5 (Fig. 6A). The Yl*rim101*Δ *mhy1*Δ mutant also resembled the *mhy1*Δ mutant in cell morphology. The induction of filament formation by the constitutively active YlRim101^1-330^ mutant at pH 4.0 also completely depends on Mhy1 (Fig. 6B). These results suggest that Mhy1 is essential for both alkaline- and glucose-induced filamentation. On the other hand, Mhy1 overexpression in both the wild-type and Yl*rim101*Δ strains caused the formation of long filaments in the yeast-form-favoring glycerol medium at pH 4.0 (Fig. 6C, top row). It also caused strong filamentation in the Yl*rim101*Δ mutant at pH 7.5 (Fig. 6C, bottom row), indicating that Mhy1 is capable of causing filamentation irrespective of the pH or the presence of glucose when its expression increases. These results suggest that Mhy1 is a key positive regulator of both alkaline- and glucose-induced filamentation.

### Mhy1 and YlRim101 positively coregulate five adhesin-like genes, and three of them appear to promote filamentation.

**FIG 7:**
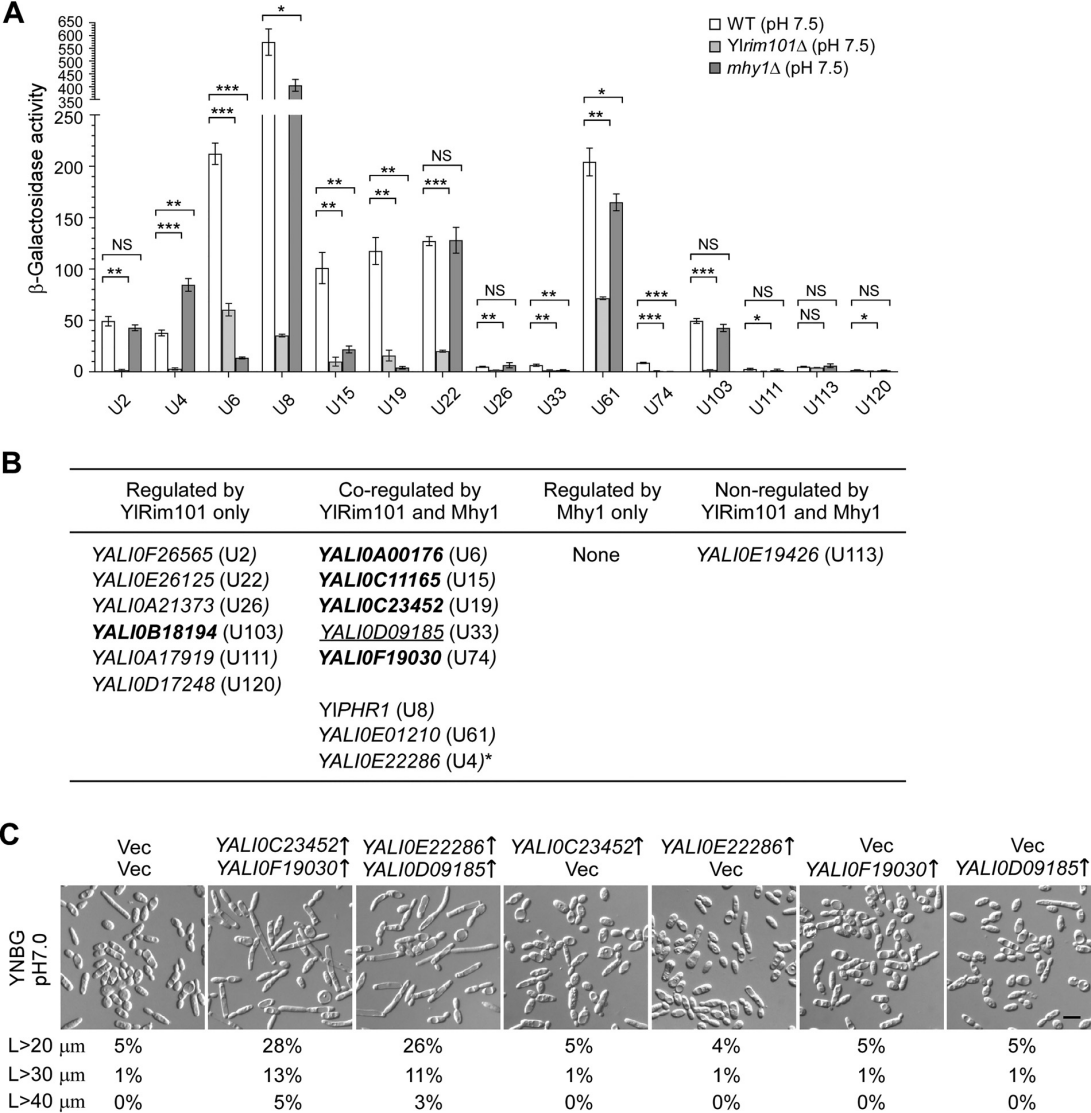
Mhy1 and YlRim101 positively coregulate five adhesin-like genes, and three of them appear to promote filamentation. (A) β-Galactosidase activities of *promoter-lacZ* for the indicated 15 highly alkaline-upregulated cell wall protein genes in cells of the wild-type, Yl*rim101*Δ, and *mhy1*Δ strains carrying pINA445-P_Gene_-*lacZ* grown in YNBG medium buffered at pH 7.5 and 30°C. Statistically significant differences are indicated by the asterisks (*, *P* < 0.05; **, *P* < 0.01; ***, *P < *0.001). NS, not statistically significant. (B) A table that lists the genes regulated by YlRim101, Mhy1, or both among the 15 highly alkaline-upregulated cell wall protein genes. The number in parentheses indicates the ranking of that gene in the full list of all alkaline-upregulated (U) genes. The genes that encode proteins that share similarities to the cell surface adhesins S. cerevisiae Flo11 and C. albicans Hyr1 are in boldface. *YALI0D09185*, which encodes a protein that shares weak similarity to the S. cerevisiae adhesin Aga1 but lacks an identifiable GPI modification site, is underlined. *, Mhy1 negatively regulates its expression. (C) Cells of the wild-type strain carrying the pair of plasmids pYL13 (Vec)/pYL21 (Vec), pYL13-YALI0C23452/pYL21-YALI0F19030, pYL13-YALI0E22286/pYL21-YALI0D09185, pYL13-YALI0C23452/pYL21, pYL13-YALI0E22286/pYL21, pYL13/pYL21-YALI0F19030, and pYL13/pYL21-YALI0D09185 were grown in YNBG medium buffered at pH 7.0. The percentages of cells longer than 20 μm, 30 μm, and 40 μm are indicated below the pictures (*n *> 600 cells). Note that the presence of *URA3*-marked vector (pYL21) decreased the lengths of elongated cells and filaments in the strain carrying *LEU2*-marked vector due to unknown reasons. Bar, 10 μm.

Alkaline pH highly upregulates the transcription of 15 cell wall protein genes (Table 1), which may play roles in filamentation. Since 14 of these genes are YlRim101 regulated, we wanted to know whether Mhy1 also regulates their expression. To this end, we monitored the transcriptional activities of the 15 cell wall protein genes in the wild-type, Yl*rim101*Δ, and *mhy1*Δ strains grown in glycerol medium at pH 7.5 by *promoter-lacZ* reporters. The results showed that eight genes exhibited differential expression in the *mhy1*Δ mutant. Of these, the five genes *YALI0A00176* (U6), *YALI0C11165* (U15), *YALI0C23452* (U19), *YALI0D09185* (U33), and *YALI0F19030* (U74) exhibited a drastic reduction by more than 3.9-fold in transcription in the *mhy1*Δ mutant (Fig. 7A), suggesting that Mhy1 is crucial for their expression. The two genes Yl*PHR1* (U8) and *YALI0E01210* (U61) also exhibited a significant reduction but to a lesser extent (1.4-fold and 1.2-fold), suggesting that Mhy1 is partly required for their expression. The remaining one, *YALI0E22286* (U4), is the only gene that exhibited an increase, which is about 2-fold, but not a reduction in the *mhy1*Δ mutant, suggesting that it was negatively regulated by Mhy1. Thus, YlRim101 and Mhy1 coregulate eight of the 15 highly alkaline-induced cell wall protein genes and positively coregulate seven of them (Fig. 7B).

To investigate whether the upregulation of alkaline-upregulated cell wall protein genes is sufficient to cause filamentation, we overexpressed 14 of the 15 highly alkaline-upregulated cell wall protein genes, except *YALI0E19426* (U113), under the control of the strong Yl*TEF1* promoter. None of these genes, including Yl*PHR1*, enhanced filament formation when overexpressed individually in the wild-type strain grown in glycerol medium at pH 7.0 (data not shown). We then overexpressed the top six of the 14 genes plus two additional YlRim101- and Mhy1-coregulated genes, *YALI0D09185* (U33) and *YALI0F19030* (U74), in pairs. The result showed that, among the 28 pairs of genes, only the two pairs *YALI0C23452* (U19)-*YALI0F19030* (U74) and *YALI0E22286* (U4)-*YALI0D09185* (U33) markedly increased the cell length upon overexpression in the wild-type strain grown in glycerol medium at pH 7.0 (Fig. 7C and data not shown), suggesting that the four genes promote filament formation. The gene *YALI0E22286* encodes a protein that shares similarity to the S. cerevisiae cell wall protein Cwp1. Interestingly, the three genes *YALI0C23452*, *YALI0F19030*, and *YALI0D09185* encode proteins that share similarities to the S. cerevisiae cell surface adhesins that mediate cell adhesion. Of these, the two genes *YALI0C23452* and *YALI0F19030* encode proteins that share similarities to the S. cerevisiae flocculin Flo11 with features including a signal peptide at the N terminus, Pro/Ser/Thr-rich repeats in the central region, potential N-glycosylation sites in the central region, and a potential GPI modification site at the C terminus, whereas the gene *YALI0D09185* encodes a protein that shares weak similarity to the S. cerevisiae a-agglutinin Aga1. Like Aga1, YALI0D09185 has a signal peptide at the N terminus and a Ser/Thr-rich central region. However, it lacks an identifiable GPI modification site at the C terminus.

Remarkably, the two YlRim101- and Mhy1-coregulated genes *YALI0A00176* and *YALI0C11165* also encode proteins that share similarities to the yeast adhesins. YALI0A00176 shares similarities to S. cerevisiae Flo11, whereas YALI0C11165 shares similarities to the C. albicans adhesin Hyr1, with features including a signal peptide at the N terminus, a Ser/Thr-rich region in the central region, a number of NNGS or NGNGS repeats that are potential N-glycosylation sites, and a potential GPI modification site at the C terminus. Together, our results indicate that Mhy1 and YlRim101 positively coregulate five adhesin-like genes, three of which appear to promote filamentation.

## DISCUSSION

Alkaline pH influences a number of cellular processes, such as nutrient uptake and protease production, in fungi. In dimorphic fungi, alkaline pH also affects the yeast-to-filament transition. While a number of cellular responses to alkaline pH are known to be regulated by the Rim101/PacC signaling pathway, the regulatory mechanism that governs alkaline pH-regulated filamentation is not well understood, except in the yeast C. albicans. In C. albicans, the Rim101/PacC signaling pathway plays an essential role in alkaline-regulated filamentation (17). However, the functional conservation of this signaling pathway is not clear in other dimorphic fungi. In this study, we show that YlRim101 is crucial for alkaline-induced filamentation in Y. lipolytica, a yeast species distantly related to C. albicans, suggesting that the Rim101/PacC signaling pathway plays a conserved role in the control of this process in dimorphic fungi. In addition to YlRim101, we identified an Msn2/Msn4-like transcription factor that is also pH responsive and essential for filamentation at alkaline pH, but its role is not shared with the Mhy1 homolog in C. albicans, suggesting that additional regulation by other factors also exists in other dimorphic fungi. Furthermore, we identified several cell wall protein genes that are coregulated by YlRim101 and Mhy1 and important for cell wall organization and filamentation.

The role of YlRim101 in the control of alkaline-regulated filamentation is largely conserved in Y. lipolytica compared to that of CaRim101 in C. albicans. YlRim101 and CaRim101 both promote alkaline-induced filamentation. In C. albicans, CaRim101 positively regulates the cell surface glycosidase genes Ca*PHR1* and Ca*CRH11* (8, 18). We find that YlRim101 also positively regulates Yl*PHR1* and Yl*CRH12*, homologs of C. albicans Ca*PHR1* and Ca*CRH11*, respectively. More importantly, the inverted pattern of CaRim101-dependent regulation on a pair of alkaline and acidic glycosidase genes, Ca*PHR1* and Ca*PHR2*, the requirement for Ca*PHR1* in cell wall organization and filamentation, as well as the requirement for Ca*PHR2* in cell wall organization in C. albicans are well conserved in Y. lipolytica. However, there are still some differences in functions between YlRim101 and CaRim101. For example, CaRim101 regulates the alkaline-induced gene Ca*KRE6* (8), which encodes a subunit of β-1,6-glucan synthase involved in cell wall biosynthesis (32). In contrast, YlRim101 does not regulate Yl*KRE6*, although it is still alkaline induced. The two yeasts also show some differences in the effectors that regulate alkaline-induced filamentation. Apart from YlRim101, the transcription factor Mhy1 plays an additional role in the regulation of alkaline-induced filamentation. However, the C. albicans homolog of Mhy1, MnlI, is reported to regulate the response to weak acid. It neither is alkaline induced nor regulates filamentation (33, 34).

Y. lipolytica can efficiently utilize both glucose and glycerol. A distinct feature of the Y. lipolytica strain PO1a is that the cells display an elongated, rod-like morphology in glucose medium but not in glycerol medium. This phenomenon is not common in other dimorphic yeasts, including C. albicans. We propose that the elongated morphology seen in cells grown in glucose medium represent an early state of filament development during the yeast-to-filament transition based on three observations. First, this morphology can be observed in wild-type cells grown in glycerol medium at pH 7.0 (Fig. 1A, top row). When the pH increases to 7.5, long filaments (hyphae) start to form. Moreover, cells expressing the constitutively active YlRim101^1-330^ mutant displayed elongated morphology in glycerol medium at acidic pH but formed long filaments at pH 7.0 (Fig. 1D, bottom row). Second, the deletion of *MHY1* abolished this morphology in glucose medium at pH 4.0 (Fig. 6A). Third, we reported previously that the inactivation of the TORC1-Sch9 signaling pathway caused the same elongated morphology and the upregulation of *MHY1* in cells grown in glycerol medium (35). Thus, it appears that glucose can stimulate filamentation, but only weakly. To date, how glucose induces filamentation remains elusive.

YlRim101 is best known for its function in the induction of the alkaline protease gene *XPR2* (24, 27). Its role in alkaline-induced filamentation has been investigated before but was underestimated (22, 23). One reason is that the culture media that were utilized to grow the yeast strains in previous studies contain filamentation-stimulating nutrients such as glucose, GlcNAc, or peptone. The Yl*rim101*Δ mutant still responded to these stimuli irrespective of the pH and might exhibit an elongated morphology or form short filaments depending on the nutrients utilized (see the bottom two rows of Fig. 1A, for example), which might have obscured the filamentation defect of the Yl*rim101*Δ mutant. Another reason appears to be the utilization of culture media buffered at pH 7.0 instead of pH 7.5 in previous studies. Since pH 7.0 induces filamentation less strongly than pH 7.5 does (see Fig. 1A, for example), the defect of the Yl*rim101*Δ mutant might be less obvious.

**FIG 8:**
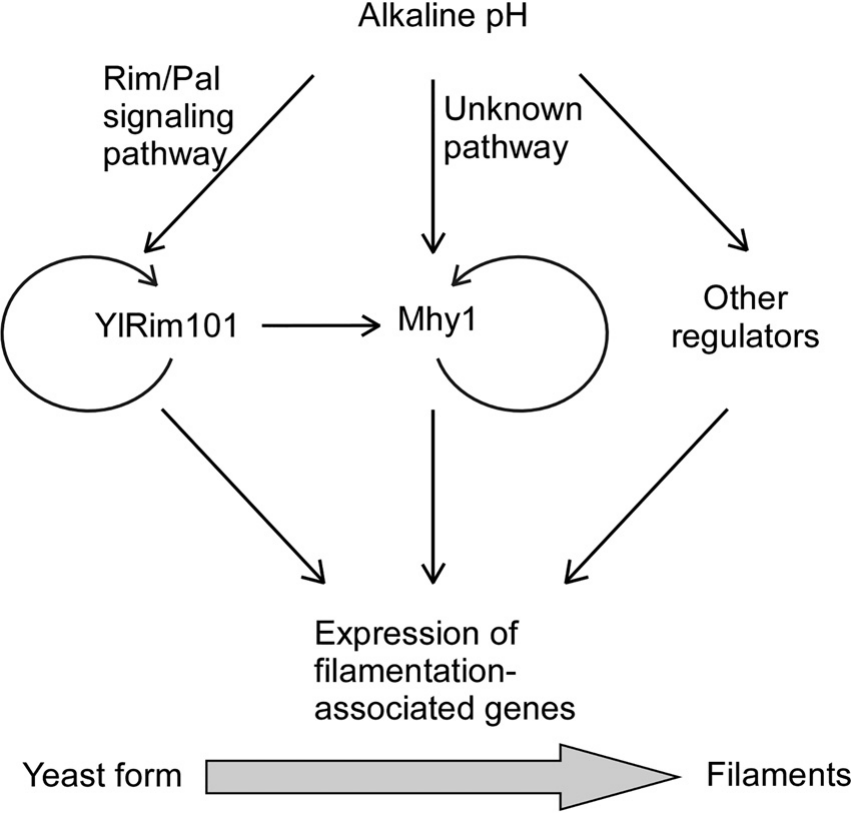
Model illustrating the regulatory mechanism that governs alkaline-induced filamentation in Y. lipolytica. YlRim101 and Mhy1 are two major effectors of alkaline pH in the regulation of alkaline-induced filamentation. They both autoregulate themselves transcriptionally. YlRim101 controls the expression of the majority of alkaline-regulated cell wall protein genes. Mhy1 and YlRim101 coregulate several filamentation-associated cell wall protein genes, including the cell surface glycosidase gene Yl*PHR1* and the five adhesin-like genes.

Based on our results, we propose a model to explain how alkaline pH induces filamentation in Y. lipolytica (Fig. 8). We propose that alkaline-induced filamentation is primarily regulated by the transcription factors YlRim101 and Mhy1. Of the two, YlRim101 is the major regulator of this process, since the majority of alkaline-upregulated cell wall protein genes and nearly all 15 highly alkaline-upregulated cell wall protein genes are YlRim101 regulated. In addition, YlRim101 partly regulates the transcription of *MHY1*. Like C. albicans CaRim101 (8), YlRim101 can also function as a repressor of gene expression, since it is involved in the downregulation of a subset of cell wall protein genes, including Yl*PHR2* (Table 1). Although we cannot rule out the possibility that some of the YlRim101-downregulated cell wall proteins are important for filamentation, we show that one of these genes, Yl*PHR2*, is not required for filamentation at alkaline pH (pH 7.5). We speculate that the activator role of YlRim101 in the regulation of cell wall protein genes is more important for YlRim101 in promoting filamentation.

Compared to YlRim101, Mhy1 appears to play a less important role in the regulation of alkaline-induced filamentation, since Mhy1 is required for the upregulation of just seven of the 15 highly alkaline-upregulated cell wall protein genes. However, we observed that *MHY1* deletion and *MHY1* overexpression affected filamentation much more strongly than those of Yl*RIM101* in glycerol medium, indicating that Mhy1 is more potent than YlRim101 in the regulation of filamentation. These two observations seem to be at odds. We hypothesize that Mhy1 also regulates some other genes that are important for filamentation but do not exhibit significant upregulation at alkaline pH. This can explain why *MHY1* deletion and *MHY1* overexpression produce a stronger phenotype. The observation that the deletion of *MHY1* abolished filamentation in glucose medium at both pH 4.0 and pH 7.5 supports this possibility. The failure of these Mhy1-regulated genes to become alkaline-upregulated might be due to other regulators of alkaline-induced filamentation that dampen the effect of Mhy1 (see below).

In addition to YlRim101 and Mhy1, other effectors that regulate alkaline-induced filamentation may also exist, because 19 of the 41 alkaline-upregulated cell wall protein genes are non-YlRim101 regulated (Table 1), and one of the 15 highly alkaline-upregulated cell wall protein genes is neither YlRim101 regulated nor Mhy1 regulated (Fig. 7B). These regulators may collectively play a minor role in the regulation of alkaline-induced filamentation. Some of these regulators may even dampen the effect of Mhy1 on the upregulation of filamentation-associated genes via downregulating the transcription levels of these genes, preventing these Mhy1-regulated genes from getting upregulated at alkaline pH. We noticed that the transcription factor gene Yl*TEC1*, which is known to repress filamentation (36), was upregulated 2-fold at pH 7.5, as shown by RNA-Seq (see Table S4 in the supplemental material).

At alkaline pH, the conserved Rim/Pal signaling pathway activates YlRim101, which in turn stimulates its own transcription, leading to a rapid accumulation of active YlRim101 in the cells. Like Yl*RIM101*, *MHY1* is highly upregulated transcriptionally at alkaline pH. This process involves both YlRim101 and an unknown YlRim101-independent mechanism. It is not known how Mhy1 is activated and by what signal. Mhy1 may need to be activated via a certain type of posttranslational modification, similar to YlRim101. It is interesting that Mhy1 stimulates its own transcription, as does YlRim101 (31). The autoregulation of YlRim101 and Mhy1 may ensure a rapid adaptation to alkaline pH for the cells.

In the dimorphic yeasts S. cerevisiae and C. albicans, the signaling pathways that promote filamentation induce the expression of several genes that encode GPI-anchored cell surface glycoproteins, such as the S. cerevisiae gene *FLO11* and the C. albicans genes *HYR1* and *HWP1* (6, 20, 37). The proteins encoded by these genes lack enzymatic activities. Some of them play an important role in filament formation and/or function as cell surface adhesins that regulate cell adhesion (21, 38–43). In C. albicans, CaRim101 upregulates the adhesin genes *HWP1* and *HYR1* at alkaline pH (8). We find that, in Y. lipolytica, among the 15 highly alkaline-upregulated cell wall protein genes, YlRim101 and Mhy1 coregulate eight of them, including the five adhesin-like genes and the cell surface glycosidase gene Yl*PHR1*. The cellular roles of these adhesin-like genes have not been established previously. The observation that three of them, *YALI0C23452*, *YALI0F19030*, and *YALI0D09185*, weakly caused cell elongation upon overexpression supports the idea that these adhesin-like genes promote filamentation.

The cell surface glycosidase gene Yl*PHR1* is required for cell wall organization and filamentation. It may also be required for cell adhesion, since C. albicans Ca*PHR1* is known to be required for cell adhesion (19). The *CRH* family genes, which encode another family of GPI-anchored cell surface glycosidases, are also required for cell wall assembly and cell adhesion in C. albicans (44, 45). We find that the three *CRH* family genes Yl*CRH11*, Yl*CRH12*, and Yl*UTR2* are all upregulated at alkaline pH. In addition, YlRim101 positively regulates Yl*CRH12*. This finding suggests that the *CRH* family genes also are required for cell wall organization and cell adhesion in Y. lipolytica.

Mhy1 is reported to bind to the DNA motif WNAGGGG (W = A or T; N = A, T, G, or C) (31). We found that all eight YlRim101- and Mhy1-coregulated cell wall protein genes contain this motif (mostly 3 to 7 copies) in the upstream intergenic region. At least one copy of this motif is present within the 1,000-bp sequence upstream of the start codon of each gene, suggesting that Mhy1 directly regulates the expression of these genes. YlRim101 is thought to bind to the DNA sequence with core motif GCCARG (R = A or G) (24), which is identical to the core consensus site of PacC in A. nidulans (9). We found that seven of the eight YlRim101- and Mhy1-coregulated cell wall protein genes, except *YALI0D09185* (U33), contain this motif (3 to 7 copies) in the upstream intergenic region. Among these seven genes, at least one copy of this motif is present within the 1,000-bp sequence upstream of the start codon in five genes. One copy of this motif is present within the 1,010-bp upstream sequence of *YALI0A00176* (U6) and within the 1,092-bp upstream sequence of *YALI0E22286* (U4). Thus, it is possible that YlRim101 directly regulates the expression of these seven genes.

The Rim101/PacC signaling pathway is evolutionarily conserved in fungi. More importantly, Rim101/PacC can function as both an activator and a repressor in the control of gene expression (9), which provides flexibility in the control of target gene expression. We hypothesize that the Rim101/PacC homologs also control pH-regulated filamentation in other dimorphic fungi.

## MATERIALS AND METHODS

### Strains and media.

The Y. lipolytica strains used in this study are listed in Table S7 in the supplemental material. PO1a (*MAT***a** *leu2-270 ura3-302*) was used as the wild-type strain. Y. lipolytica strains were grown at 30°C in YPD medium (20 g/liter peptone, 10 g/liter yeast extract, 2% glucose), YNBD medium (6.7 g/liter yeast nitrogen base without amino acid, 1% glucose), or YNBG medium (6.7 g/liter yeast nitrogen base without amino acid, 1% glycerol) supplemented with 80 mg/liter leucine, 20 mg/liter uracil, or both, when required. YNBD and YNBG media were buffered after autoclave to pH values ranging from 3.0 to 7.5 with Na_2_HPO_4_–citric acid buffer. The Escherichia coli strain DH5α was used for plasmid amplification.

10.1128/mSphere.00179-21.7 TABLE S6 RNA-Seq data for transcription factor genes in Yl*rim101*Δ (pH 4.0) versus WT (pH 4.0) datasets. Download Table S6, XLS file, 0.04 MB. Copyright © 2021 Shu et al. 2021 Shu et al. https://creativecommons.org/licenses/by/4.0/ This content is distributed under the terms of the Creative Commons Attribution 4.0 International license.

10.1128/mSphere.00179-21.8 TABLE S7 Yarrowia lipolytica strains used in this study. Download Table S7, DOCX file, 0.02 MB. Copyright © 2021 Shu et al. 2021 Shu et al. https://creativecommons.org/licenses/by/4.0/ This content is distributed under the terms of the Creative Commons Attribution 4.0 International license.

### Plasmid construction.

The plasmids used in this study are listed in Table S8. To generate the plasmid pINA445-YlRIM101, the Yl*RIM101* gene, which contains the 2,946-bp promoter and 282-bp 3′-untranslated region (UTR), was amplified by PCR and inserted into ClaI- and HindIII-digested vector pINA445 (*CEN* and *LEU2*). The Yl*RIM101^1-419^* and Yl*RIM101^1-330^* mutant genes carrying the 2,946-bp promoter and 282-bp 3′-UTR were generated from pINA445-YlRIM101 by overlapping PCR and inserted into pINA445, yielding pINA445-YlRIM101^1-419^ and pINA445-YlRIM101^1-330^. Yl*RIM101^1-330^* was also inserted into pINA443 (*CEN*, *URA3*), yielding pINA443-YlRIM101^1-330^. The Yl*PHR1* gene, which contains the 1,995-bp promoter and 960-bp 3′-UTR, was amplified by PCR and inserted into pINA445 using the ClonExpress II one-step cloning kit (Vazyme Biotech Co., China), yielding pINA445-YlPHR1. Similarly, the Yl*PHR2* gene, which contains a 1,992-bp promoter and 960-bp 3′-UTR, was amplified by PCR and inserted into pINA445.

10.1128/mSphere.00179-21.9 TABLE S8 Plasmids used in this study. Download Table S8, DOCX file, 0.03 MB. Copyright © 2021 Shu et al. 2021 Shu et al. https://creativecommons.org/licenses/by/4.0/ This content is distributed under the terms of the Creative Commons Attribution 4.0 International license.

To monitor the transcriptional activities of the cell wall protein genes that were highly upregulated at pH 7.5, the promoter region of each gene plus the ATG start codon was amplified by PCR and inserted into pINA445-*lacZ* (36) using the ClonExpress II one-step cloning kit, yielding pINA445-P_Gene_-*lacZ*. pINA445-PYlRIM101-*lacZ* and pINA445-PYlPHR2-*lacZ* were generated similarly. pINA445-P_MHY1_-*lacZ* carrying the 4,308-bp *MHY1* promoter was described previously (31).

To overexpress the cell wall protein genes driven by the Yl*TEF1* promoter, the open reading frame (ORF) of each gene plus the 3′-UTR was amplified by PCR and inserted into pYL13 (*CEN*, *LEU2*, and *P_YlTEF1_*) (36), yielding pYL13-Gene. These genes were also inserted into pYL21 (*CEN*, *URA3*, and *P_YlTEF1_*), yielding pYL21-Gene. To generate pYL21, a DNA fragment that contains the Yl*TEF1* promoter, multiple cloning sites from XbaI to KpnI as in pBlueScript KS (+), and the Yl*URA3* gene was ligated with the EcoRI/BglII-digested large fragment of pINA445, which lacks the Yl*LEU2* gene and *Tet^R^* gene. The EcoRI site was destroyed and replaced by an NcoI site. pYL13-MHY1 was described previously (31).

### Yeast strain construction.

DNA was transformed into Y. lipolytica cells by the lithium acetate method (36). Yl*RIM101* was deleted in the wild-type strain PO1a by homologous recombination. Briefly, an ∼1.0-kb sequence upstream of the Yl*RIM101* ORF (RIM101P) and an ∼1.0-kb sequence downstream of the ORF (RIM101T) were amplified by PCR from genomic DNA. RIM101P and RIM101T were then inserted into the flanking sites of *loxR*-Yl*URA3*-*loxP* in pYL8 (36). The resulting RIM101P-loxR-YlURA3-loxP-RIM101T deletion cassette was used to transform strain PO1a. Ura^+^ transformants were examined by PCR to identify the correct Yl*rim101*Δ::*loxR*-Yl*URA3*-*loxP* clones. The Yl*URA3* marker was later excised by Cre-mediated site-specific DNA recombination between *loxR* and *loxP* sites (36), yielding the Yl*rim101*Δ::*loxR-P* strain. The Yl*rim101*Δ *mhy1*Δ, Yl*phr1*Δ, Yl*phr2*Δ, and Yl*phr1*Δ Yl*phr2*Δ mutants were constructed similarly. The strain YLX497 (*mhy1*Δ) was described previously (31).

### RNA-Seq analysis.

Y. lipolytica cells were grown in liquid YNBG medium supplemented with uracil and buffered at pH 7.5 or pH 4.0 at 30°C. Cells were harvested when the optical density at 600 nm (OD_600_) reached 1.0. Three replicates were performed for each sample. Total RNA was extracted using a yeast RNA kit (Omega, China) by following the manufacturer’s instructions. A total amount of 1 μg RNA per sample was used. Sequencing libraries were generated using a TruSeq RNA library preparation kit (Illumina, USA). Paired 150-bp sequencing was performed on a NovaSeq 6000 (Illumina) at Berry Genomics Corporation (Beijing, China). The RNA-Seq reads were checked for quality by FastQC before cleaning and by Trimmomatic after cleaning and were then mapped to the reference genome (Y. lipolytica CLIB122) using HISAT2 (v2.0.6) (46). Raw read counts for each gene were calculated using HTSeqCount (47). Read count data were normalized using edgeR (R-3.3.3) (48). Normalized read counts were used to identify differentially expressed genes with adjusted *P* values of <0.05 and fold change below −2.0 or above 2.0.

### β-Galactosidase assay.

The β-galactosidase activity in the cells was determined by the crude cell extract assay with o-nitrophenyl-β- D –galactopyranoside (ONPG) as the substrate, as reported previously (36). Crude cell extracts were prepared by vortexing with glass beads. Protein concentration in the cell extracts was measured by the Bradford method. The specific β-galactosidase activity was normalized by the amount of total protein in each extract and was calculated according to the following formula: U = (OD_420_ × 1.7)/[0.0045 × protein concentration (mg ml^−1^) × sample volume (ml) × time (min)]. The assays were performed in triplicate.

### Microscopy.

Y. lipolytica cells were grown in liquid YNBG or YNBD medium for 16 h at 30°C before differential interference contrast (DIC) images were taken. An Olympus BX51 microscope (Tokyo, Japan) and a Retiga 2000R charge-coupled device (CCD) camera (QImaging Corporation, Canada) were used to visualize cell morphology. The DIC images were acquired using QCapture Suite (QImaging Corporation, Canada).

### Data availability.

RNA-Seq data can be found in the tables in the supplemental material. Full data can be obtained from the authors upon request.
