# Supplementary figures and images for: The pH-Responsive Transcription Factors YlRim101 and Mhy1 Regulate Alkaline pH-Induced Filamentation in the Dimorphic Yeast Yarrowia lipolytica
Source: mSphere. 2021 May 19;6(3):e00179-21. doi: 10.1128/mSphere.00179-21 (PMC8265631; doi:10.1128/mSphere.00179-21)

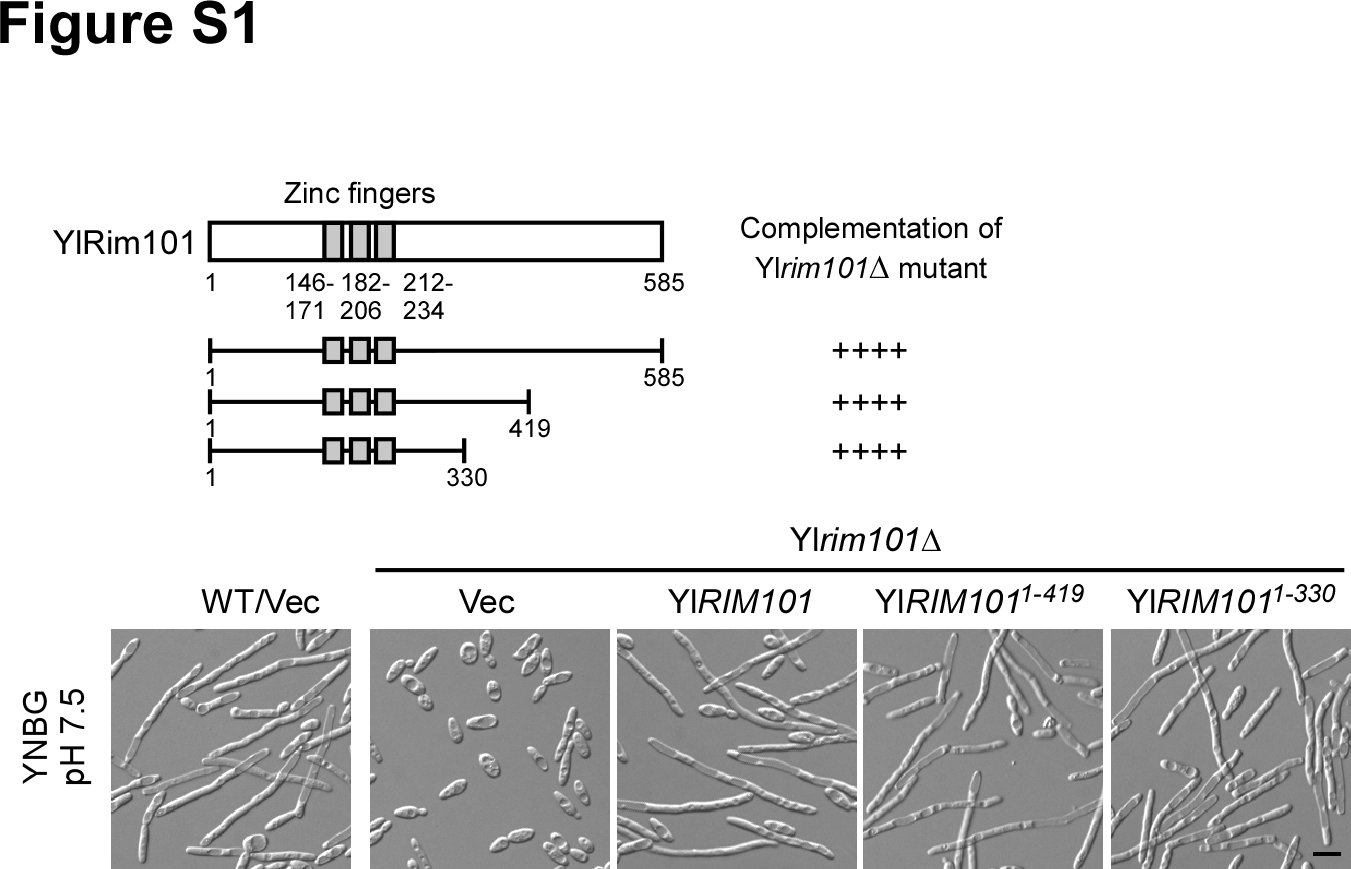

Supplement: FIG S1 [file msphere.00179-21-sf001.tif]
