# Supplementary material for: The pH-Responsive Transcription Factors YlRim101 and Mhy1 Regulate Alkaline pH-Induced Filamentation in the Dimorphic Yeast Yarrowia lipolytica
Source: mSphere. 2021 May 19;6(3):e00179-21. doi: 10.1128/mSphere.00179-21 (PMC8265631; doi:10.1128/mSphere.00179-21)
Supplement: TABLE S7 [file msphere.00179-21-st007.docx]

**Table S7. *Yarrowia lipolytica* strains used in this study.**

| **Strain** | **Genotype** | **Source** |
| --- | --- | --- |
| PO1a | *MATA leu2-270 ura3-302* | 1 |
| YLX497 | As PO1a except *mhy1*Δ*::loxR/P* | 2 |
| YLX514 | As PO1a except Yl*rim101*Δ*::loxR/P* | This study |
| YLX515 | As PO1a except Yl*rim101*Δ*::loxR/P mhy1*Δ*::loxR/P* | This study |
| YLX516 | As PO1a except Yl*phr1*Δ*::loxR/P* | This study |
| YLX517 | As PO1a except Yl*phr2*Δ*::loxR/P* | This study |
| YLX518 | As PO1a except Yl*phr1*Δ*::loxR/P* Yl*phr2*Δ*::loxR/P* | This study |
|  | **References** | |
| 1 | Barth G and Gaillardin C (1996) The dimorphic fungus *Yarrowia lipolytica*. p.313-368. In K. Wolf (ed). Non-conventional yeasts in biotechnology. Springer, Heidelberg, Germany. | |
| 2 | Wu H, Shu T, Mao Y-S and Gao X-D (2020) Characterization of the promoter, downstream target genes and recognition DNA sequenced of Mhy1, a key filamentation-promoting transcription factor in the dimorphic yeast *Yarrowia lipolytica*. *Curr. Genet*. 66: 245-261. | |
