# Supplementary material for: The pH-Responsive Transcription Factors YlRim101 and Mhy1 Regulate Alkaline pH-Induced Filamentation in the Dimorphic Yeast Yarrowia lipolytica
Source: mSphere. 2021 May 19;6(3):e00179-21. doi: 10.1128/mSphere.00179-21 (PMC8265631; doi:10.1128/mSphere.00179-21)
Supplement: TABLE S8 [file msphere.00179-21-st008.docx]

**Table S8. Plasmids used in this study.**

| **Plasmid** | **Description** | **Source** |
| --- | --- | --- |
| pINA445 | pBR322 carrying *ARS68 (CEN/ARS)* and Yl*LEU2* | 1 |
| pINA443 | pBR322 carrying *ARS68 (CEN/ARS)* and Yl*URA3* | Richard Rachubinski |
| pINA445-YlRIM101 | Yl*RIM101* with 2946-bp promoter and 282-bp 3’-UTR | This study |
| pINA445-YlPHR1 | Yl*PHR1* with 1995-bp promoter and 960-bp 3’-UTR | This study |
| pINA445-YlPHR2 | Yl*PHR2* with 1992-bp promoter and 960-bp 3’-UTR | This study |
| pINA445-YlRIM101^1-419^ | Yl*RIM101^1-419^* encoding a.a. 1-419 with 2946-bp promoter and 282-bp 3’-UTR | This study |
| pINA445-YlRIM101^1-330^ | Yl*RIM101^1-330^* encoding a.a. 1-330 with 2946-bp promoter and 282-bp 3’-UTR | This study |
| pINA443-YlRIM101^1-330^ | Yl*RIM101^1-330^* encoding a.a. 1-330 with 2946-bp promoter and 282-bp 3’-UTR | This study |
| pINA445-lacZ | 3.3 kb *lacZ* gene from plasmid pSH18-34 | 2 |
| pINA445-P_U2_-lacZ | 2703-bp *YALI0F26565* promoter fused to *lacZ* | This study |
| pINA445-P_U4_-lacZ | 3819-bp *YALI0E22286* promoter fused to *lacZ* | This study |
| pINA445-P_U6_-lacZ | 4299-bp *YALI0A00176* promoter fused to *lacZ* | This study |
| pINA445-P_U8_-lacZ (pINA445-P_YlPHR1_-lacZ) | 4794-bp Yl*PHR1* (*YALI0D04851*) promoter fused to *lacZ* | This study |
| pINA445-P_U15_-lacZ | 3819-bp *YALI0C11165* promoter fused to *lacZ* | This study |
| pINA445-P_U19_-lacZ | 2748-bp *YALI0C23452* promoter fused to *lacZ* | This study |
| pINA445-P_U22_-lacZ | 2637-bp *YALI0E26125* promoter fused to *lacZ* | This study |
| pINA445-P_U26_-lacZ | 3255-bp *YALI0A21373* promoter fused to *lacZ* | This study |
| pINA445-P_U33_-lacZ | 1260-bp *YALI0D09185* promoter fused to *lacZ* | This study |
| pINA445-P_U61_-lacZ | 2919-bp *YALI0E01210* promoter fused to *lacZ* | This study |
| pINA445-P_U74_-lacZ | 5688-bp *YALI0F19030* promoter fused to *lacZ* | This study |
| pINA445-P_U103_-lacZ | 2262-bp *YALI0B18194* promoter fused to *lacZ* | This study |
| pINA445-P_U111_-lacZ | 5595-bp *YALI0A17919* promoter fused to *lacZ* | This study |
| pINA445-P_U113_-lacZ | 3630-bp *YALI0E19426* promoter fused to *lacZ* | This study |
| pINA445-P_U120_-lacZ | 1380-bp *YALI0D17248* promoter fused to *lacZ* | This study |
| pINA445-P_YlPHR2_-lacZ | 3282-bp Yl*PHR2* promoter fused to *lacZ* | This study |
| pINA445-P_MHY1_-lacZ | 4308-bp *MHY1* promoter fused to *lacZ* | 3 |
| pINA445-P_YlRIM101_-lacZ | 5460-bp *YlRIM101* promoter fused to *lacZ* | This study |
| pYL13 | *ARS68 (CEN/ARS)*, Yl*LEU2*, *P_YlTEF1_* | 2 |
| pYL13-YALI0C23452 | *P_YlTEF1_-YALI0C23452* plus 860-bp 3’-UTR in pYL13 | This study |
| pYL13-YALI0E22286 | *P_YlTEF1_-YALI0E22286* plus 1000-bp 3’-UTR in pYL13 | This study |
| pYL13-MHY1 | *P_YlTEF1_-MHY1* plus 327-bp 3’-UTR in pYL13 | 3 |
| pYL21 | *ARS68 (CEN/ARS)*, Yl*URA3*, *P_YlTEF1_* | This study |
| pYL21-YALI0D09185 | *P_YlTEF1_-YALI0D09185* plus 150-bp 3’-UTR in pYL21 | This study |
| pYL21-YALI0F19030 | *P_YlTEF1_-YALI0F19030* plus 1000-bp 3’-UTR in pYL21 | This study |
| pYL8 | pBlueScript KS(+) carrying *loxR*-Yl*URA3*-*loxP* | This study |
| pYL8-YlRIM101 | *P_YlRIM101_*-*loxR*-Yl*URA3*-*loxP*-*T_YlRIM101_* in pYL8 | This study |
| pYL8-MHY1 | *P_MHY1_*-*loxR*-Yl*URA3*-*loxP*-*T_MHY1_* in pYL8 | 3 |
| pYL8-YlPHR1 | *P_YlPHR1_*-*loxR*-Yl*URA3*-*loxP*-*T_YlPHR1_* in pYL8 | This study |
| pYL8-YlPHR2 | *P_YlPHR2_*-*loxR*-Yl*URA3*-*loxP*-*T_YlPHR2_* in pYL8 | This study |
| pRRQ2 | *ARS68 (CEN/ARS)*, Yl*LEU2*, *hp4d-CRE* | 4 |
|  | **References** | |
| 1 | Nuttley WM, Brade AM, Gaillardin C, Eitzen GA, Glover JR, Aitchison JD and Rachubinski RA (1993) Rapid identification and characterization of peroxisomal assembly mutants in *Yarrowia lipolytica*. *Yeast*. 9: 507–517. | |
| 2 | Zhao X-F, Li M, Li Y-Q, Chen X-D and Gao X-D (2013) The TEA/ATTS transcription factor YlTec1p represses the yeast-to-hypha transition in the dimorphic yeast *Yarrowia lipolytica*. *FEMS Yeast Res*. 13: 50-61. | |
| 3 | Wu H, Shu T, Mao Y-S and Gao X-D (2020) Characterization of the promoter, downstream target genes and recognition DNA sequenced of Mhy1, a key filamentation-promoting transcription factor in the dimorphic yeast *Yarrowia lipolytica*. *Curr. Genet*. 66: 245-261. | |
| 4 | Richard M, Quijano RR, Bezzate S, Bordon-Pallier F and Gaillardin C (2001) Tagging morphogenetic genes by insertional mutagenesis in the yeast *Yarrowia lipolytica*. *J. Bacteriol*. 183: 3098-3107. | |
